# Knowledge and Prevalence of Vaginismus Among Women in Najran, Saudi Arabia

**DOI:** 10.7759/cureus.48602

**Published:** 2023-11-10

**Authors:** Majed S Alshahrani, Albatoul M Al Margan, Anwar M Al Sharyan

**Affiliations:** 1 Obstetrics and Gynaecology, Najran University, Faculty of Medicine, Najran, SAU; 2 College of Medicine, Najran University, Najran, SAU

**Keywords:** ksa, knowledge of vaginismus, saudi arabia, vaginismus, prevalence

## Abstract

Background: Vaginismus is a distressing sexual dysfunction that can profoundly impact women's physical and psychological well-being. Understanding its prevalence and associated factors is crucial for effective healthcare provision. This study aims to investigate the prevalence of vaginismus and assess knowledge about the condition among women in Najran, Saudi Arabia.

Methods: A cross-sectional study was conducted in May 2023. Data were collected via an online survey, with 500 adult women in Najran participating. The survey assessed demographics, vaginismus complaints, and knowledge about vaginismus. Statistical analysis included chi-square tests, logistic regression, and correlations.

Results: The prevalence of vaginismus complaints among participants was 4.6%. Age, particularly being over 45 years old, was a significant predictor of vaginismus complaints. Marital status also showed a significant association, with married women reporting a higher prevalence. BMI, health status, and various menstrual characteristics were not significant predictors. Knowledge about vaginismus was moderate, with 60% of participants lacking a source of information.

Conclusion: Vaginismus is a relatively rare but significant concern among women in Najran, Saudi Arabia, with age and marital status being notable factors. The study highlights the need for improved sexual health education and awareness, particularly among older women, to address this sensitive issue effectively. Further research should delve into the psychological and cultural factors influencing vaginismus in this population.

## Introduction

Vaginismus is characterized by an involuntary spasm of the muscles surrounding the vaginal wall, making sexual intercourse uncomfortable. Because of its large psychogenic component, this topic is frequently overlooked by professionals, despite the fact that it is extremely sensitive for female patients [1].

Healthcare professionals are confused as to why certain people have vaginismus. It can lead to physical, psychological, and sexual problems. Bladder infections, urinary tract infections, and yeast infections can aggravate vaginismus pain. Anxiety problems, birthing injuries such as vaginal rips, prior surgery, dread of sex, or unpleasant thoughts about sex, possibly owing to past sexual abuse, rape, or trauma, are all factors that may lead to vaginismus [2].

Despite its widespread occurrence, vaginismus is understudied. There are multiple interpretations of vaginismus, including the medical definition of vaginismus as a spastic reaction, which has underpinned much of the symptom-focused behavioral and cognitive therapies that have dominated the area of sex therapy so far. This symptom-focused approach has been challenged as a reductionist, with several authors advocating for a more holistic approach to understanding and treating vaginismus [3].

The attribution of the problem to psychological rather than physical factors is one of the indicators of successful treatments for vaginismus. To completely comprehend vaginismus, it must be investigated at the intrapersonal, interpersonal, and cultural levels, with the interpersonal level receiving the most attention [4]. Integrating a biopsychosocial and experiential approach into perspectives on vaginismus may aid therapists who work with individuals or couples who appear with vaginismus in their practice. To successfully respond to and overcome the complicated nature of this distressing sexual challenge for women and men in adult intimate relationships, a multidisciplinary approach to therapy of vaginismus is more suited [5].

There were similarities in aspects of sexual functions, behaviors, and cognitions between women with vaginismus and controls; women with vaginismus differed in concerns about losing control of the body and/or situation and demonstrated behaviors suggestive of greater avoidance of sexual behavior and penetration compared to the control group. The most noticeable symptoms of women with vaginismus were dread of pain and worry of having a panic attack during intercourse; nevertheless, painful intercourse (dyspareunia), while present in all groups, was more prevalent in the control group [6,7].

In Arab-Muslim civilizations, vaginismus is one of the most common causes of marriage non-conclusion and infertility. Cognitive behavioral therapy (CBT) has been shown to be successful, although it is vital to remember the patient's cultural background when attending CBT for vaginismus [8]. In general, patients do not seek out a psychotherapist for this issue; instead, they are referred by gynecologists. Inadequate sexual knowledge appears to be the most significant possible risk factor for developing this illness. Particular consideration should be given to the cultural and religious components of vaginismus in Arab-Muslim patients. Initially, vaginismus should be viewed as a family problem rather than the couple's single issue [9,10].

Integrating a biopsychosocial and experiential approach into perspectives on vaginismus may aid therapists who work with individuals or couples who appear with vaginismus in their practice. To successfully respond to and overcome the complicated nature of this distressing sexual challenge for women and men in adult intimate relationships, a multidisciplinary approach to therapy of vaginismus is more suited [11].

Velayati et al. reported that depression and anxiety were two significant variables that influenced the sexual functioning (sexual quality of life (SQOL) of research participants. As a result, it is also preferable to pay more attention to these aspects in the therapy of sexual dysfunction. The current study's findings are proposed to be used as a foundation for developing appropriate and effective interventions to prevent psychological illnesses, particularly anxiety and depression, in women with vaginismus problems [12]. Another study found that in women with vaginismus, psychological characteristics, such as anxiety, sadness, and self-esteem, are predictors of SQOL [13].

The only known study of vaginismus in Ireland in the last 40 years, however, reveals that the Catholic Church's legacy in Ireland has fostered a culture of quiet and shame around sexual problems, which may compound feelings of shame and isolation for persons with sexual disorders [14]. They also discovered that Irish women with vaginismus frequently indicated that their mothers' messages about sex and pregnancy were terrifying [14]. Gaber et al. investigated the incidence of vaginismus and its impact on human sexual life in Delta, Egypt. They found that vaginismus is a widespread and serious sexual condition that causes both couples anguish and dissatisfaction [15]. This study aims to investigate the prevalence of vaginismus among women in Najran, Saudi Arabia. Our study will also assess their knowledge of vaginismus.

## Materials and methods

Study design

This study employed a cross-sectional design to investigate the prevalence of vaginismus and assess the knowledge of vaginismus among women residing in Najran, Saudi Arabia. A cross-sectional approach was chosen to provide a snapshot of the current status of vaginismus in the study population and to collect data on associated factors.

Study setting and duration

The study was conducted in Najran, a city in the southern region of Saudi Arabia, during the month of May 2023. Najran was chosen as the study setting due to its diverse population and representation of the Saudi community.

Study participants

The study included adult females aged 18 years and above, who were married and residing in Najran, Saudi Arabia. The inclusion criteria were set to target the population most likely to be affected by vaginismus, given that it is a condition often associated with sexual activity and relationships. Participants were required to provide informed consent and agree to complete the survey.

Exclusion criteria

Individuals with cognitive impairments that hindered their ability to respond to the questionnaire were excluded from the study. Additionally, participants who refused to participate or provide informed consent were excluded.

Recruitment and data collection

The recruitment of participants was primarily conducted through social media platforms. An online self-administered questionnaire, prepared in the Arabic language, was used for data collection. The questionnaire was designed to collect data anonymously to ensure participant privacy.

Data collection tool

The online survey instrument consisted of several sections: Demographic data: This section collected information on age, nationality, marital status, level of education, and health status. Experience with vaginismus: Participants were asked whether they complained of vaginismus (yes/no). Awareness and understanding of vaginismus: This section aimed to assess participants' knowledge of vaginismus, including its causes, symptoms, and complications. Perception of vaginismus: Participants were questioned about their perceptions and attitudes toward vaginismus. Source of knowledge: Participants were asked where they obtained information about vaginismus. Diagnosis: Participants were questioned about how they were diagnosed with vaginismus.

Sample size

The sample size was estimated using the Raosoft® calculator, with a 5% level of significance, a 5% margin of error, a 95% confidence level, and an expected response distribution of 50%. The calculated sample size was 385 participants.

Data analysis

Data analysis was performed using the IBM SPSS Statistics for Windows, version 23 (released 2015; IBM Corp., Armonk, New York, United States). The collected data were coded into variables, and the normality of data was assessed using the Kolmogorov-Smirnov test. Descriptive and inferential statistics, including the chi-square test, Mann-Whitney U test, Kruskal-Wallis H test, and Spearman's correlation, were employed to present the results. A p-value of less than 0.05 was considered statistically significant.

The knowledge section was scored, with each correct answer assigned 1 mark, while wrong or "don't know" responses received 0 marks. This scoring method resulted in a total knowledge score range of 0 to 16 for the knowledge section.

Ethical considerations

The patients' confidentiality and the privacy of their data are the priority. Leads to ethical issues, such as the name of the participants, were used. The ethical approval was obtained from Kingdom of Saudi Arabia Ministry of Education Najran University Deanship of Scientific Research Scientific Research Ethical Committee (approval number 012132-027251-DS). 

## Results

Table 1 presents the demographic profile of the study participants (N=500), providing valuable insights into the characteristics of the women surveyed in Najran, Saudi Arabia.

**Table 1:** Demographic profile of the study participants (N=500)

Variables	N	%
Do you complain of vaginismus?		
No	477	95.4
Yes	23	4.6
Knowledge score (mean ± SD)	(9.84±2.094)/16 = 62.5%	
BMI (mean ± SD)	(24.94 ±6.076)	
BMI		
<18.5 underweight	49	9.8
18.5-24.9 normal weight	221	44.2
25-29.9 overweight	155	31
30-34.9 obesity class 1	57	11.4
35-39.9 obesity class 2	8	1.6
≥40 obesity class 3	9	1.8
Age		
Less than 25 years	241	48.2
From 26-45 years	226	45.2
Over 45 years	33	6.6
Nationality		
Saudi	471	94.2
Non-Saudi	29	5.8
Marital status		
Single	267	53.4
Married	232	46.4
Level of education		
Illiterate	6	1.2
Secondary	104	20.8
University	390	78
Health status		
Healthy	470	94
Diseased	30	6
Age at the onset of menstruation		
Less than 11 years old	56	11.2
12-14	341	68.2
15 17	90	18
More than 17 years old	13	2.6
Duration of the menstrual cycle		
Less than 21 days	363	72.6
21-25 days	115	23
More than 35 days	22	4.4
Is the menstrual cycle regular?		
No	119	23.8
Yes	381	76.2
Number of days of menstrual blood		
Less than 3 days	21	4.2
From 3 to 7 days	431	86.2
More than 7 days	48	9.6
The amount of menstrual blood is abundant.		
Low	36	7.2
Medium	433	86.6
Abundant	31	6.2
Do you suffer from menstrual pain?		
No	49	9.8
Low	143	28.6
Mild	211	42.2
Severe	97	19.4
Source of knowledge		
Nil	286	57.2
Social media	109	21.8
Medical service providers	21	4.2
Central media	18	3.6
Friends and family	66	13.2
How were you diagnosed?		
Nil	478	95.6
Themselves	7	1.4
Doctor	15	3.0

Among the participants, 95.4% (n=477) reported no complaints of vaginismus, while 4.6% (n=23) indicated experiencing vaginismus symptoms. This indicates that vaginismus is a relatively uncommon issue among the surveyed women. The mean knowledge score regarding vaginismus was found to be 9.84±2.094 out of a possible 16, representing an average score of 62.5%. This suggests that, on average, the participants possess a moderate level of awareness and understanding of vaginismus causes, symptoms, and complications.

The average BMI of the participants was 24.94±6.076, falling within the normal weight range (18.5-24.9) for the majority of respondents (44.2%, n=221). A significant portion of the participants also fell into the overweight category (31%, n=155), with a smaller percentage in obesity class 1 (11.4%, n=57), obesity class 2 (1.6%, n=8), and obesity class 3 (1.8%, n=9). The age distribution of the participants revealed that 48.2% were less than 25 years old, 45.2% (n=22.6) fell within the age range of 26-45 years, and 6.6% (n=33) were over 45 years old. This distribution provides a representative sample across different age groups.

The vast majority of participants (94.2%, n=470) were of Saudi nationality, with a smaller percentage (5.8%, n=29) being non-Saudi residents in Najran. Approximately half of the participants were single (53.4%, n=267), while the other half were married (46.4%, n=232), indicating a balanced representation of marital status in the sample.

Most participants had a university-level education (78%, n=390), while a significant proportion had a secondary education (20.8%, n=104), and a small percentage were illiterate (1.2%, n=6). This diverse educational background is reflected in the sample. The majority of participants reported being in good health (94%, n=470), with a smaller percentage indicating they had a pre-existing medical condition (6%, n=30).

The participants reported varying age at the onset of menstruation, with 68.2% (n=341) experiencing it between the ages of 12-14 years. Most participants had menstrual cycles lasting less than 21 days (72.6%, n=363) and reported having regular menstrual cycles (76.2%, n=363). Regarding the number of days of menstrual bleeding, the majority (86.2%, n=341) had bleeding that lasted from three to seven days. Additionally, most participants reported medium menstrual blood flow (86.6%, n=433). The participants' experiences with menstrual pain varied, with 42.2% (n=211) reporting mild pain and 19.4% experiencing severe pain during menstruation. A smaller percentage had low pain (28.6%, n=143), and a notable portion reported no menstrual pain 9.8% (n=49).

The primary sources of knowledge about vaginismus were found to be friends and family 13.2% (n=66), social media 21.8% (n=108), and medical service providers 4.2% (n=21), while a significant proportion 57.2% (n=286) reported having no source of knowledge about vaginismus. This highlights the need for increased awareness and education on this topic. The majority of participants (95.6%, n=478) reported not receiving a formal diagnosis of vaginismus, while a small number had self-diagnosis (1.4%, n=7) or received a diagnosis from a doctor (3.0%, n=15).

Table 2 presents the correlation between knowledge scores and BMI among the study participants. A statistically significant negative correlation was observed between knowledge scores and BMI (Spearman's correlation=-0.091, p=0.042). This finding indicates that as BMI increases, knowledge about vaginismus tends to decrease slightly. It is important to note that while the correlation is statistically significant, the practical significance of this relationship may be limited due to the relatively low correlation coefficient.

**Table 2:** Correlation between the knowledge score and BMI *Significant association Spearman's correlation

P value		BMI	Knowledge score
Body mass index	Spearman's correlation	1	-.091^*^
	Sig. (2-tailed)	0.042	
	N	500	500

Table 3 explores the correlation between different variables and participants' knowledge scores regarding vaginismus. Several variables were analyzed, including complaints of vaginismus, nationality, regularity of the menstrual cycle, marital status, age, level of education, health status, age at the onset of menstruation, duration of the menstrual cycle, number of days of menstrual blood, amount of menstrual blood, suffering from menstrual pain, source of knowledge, and method of diagnosis.

**Table 3:** Correlation of different variables and the knowledge score of vaginismus P-value < 0.05 is statistically significant; * Mann-Whitney U test **Kruskal-Wallis H test

Variables	Mean rank	P-value
Do you complain of vaginismus?		
No	250.72	P-value (0.873) U **= 48765378.500
Yes	245.85	
Nationality		
Saudi	250.55	P-value (973) U **= 6804.000
Non-Saudi	249.62	
Is the menstrual cycle regular?		
No	242.05	P-value (.460) U **= 21664.000
Yes	253.14	
Marital status		
Single	244.35	P-value (.342) U *= 29462.500
Married	256.51	
Age		
Less than 25 years	240.91	P-value (.103) χ2 = 4.541
From 26 to 45 years	264.73	
Over 45 years	223.05	
Level of education		
Illiterate	388.33	P-value (.059) χ2** = 5.663
Secondary	247.09	
University	249.29	
Health status		
Healthy	249.16	P-value (0.4) U *= 6419.500
Diseased	271.52	
Age at the onset of menstruation		
Less than 11 years old	207.51	P-value (.091) χ2 = 6.455
12-14	256.28	
15-17	250.12	
More than 17 years old	286.73	
Duration of the menstrual cycle		
Less than 21 days	251.33	P-value (.963) χ2 =.076
21-25 days	247.36	
More than 35 days	253.25	
Number of days of menstrual blood		
Less than 3 days	243.38	P-value (.564) χ2 = 1.146
From 3 to 7 days	253.09	
More than 7 days	230.35	
The amount of menstrual blood is abundant.		
Low	237.56	P-value (.776) χ2 =.508
Medium	252.28	
Abundant	240.68	
Do you suffer from menstrual pain?		
No	247.34	P-value (.039) χ2 = 8.393
Low	277.96	
Mild	243.31	
Severe	227.27	
A source of knowledge		
Nil	243.12	P-value (.249) χ2 = 5.399
Social media	268.71	
Medical service providers	269.52	
Central media	294.06	
Friends and family	234.49	
How were you diagnosed?		
Nil	251.08	P-value (.828) χ2 =.376
Themselves	218.07	
Doctor	247.10	
BMI stages		
<18.5 underweight	264.44	P-value (.606) χ2 = 3.617
18.5-24.9 normal weight	256.57	
25-29.9 overweight	241.40	
30-34.9 obesity class 1	238.66	
35-39.9 obesity class 2	286.19	
≥40 obesity class 3	197.72	

No statistically significant correlation was found between knowledge scores and most of these variables, as indicated by the p-values exceeding the significance threshold of 0.05. However, it is worth noting a few exceptions. A statistically significant correlation was observed between the knowledge scores and suffering from menstrual pain (p=0.039) using the chi-square test. This suggests that the participants who reported experiencing menstrual pain tended to have different knowledge levels compared to those who did not. Additionally, a statistically significant correlation was found between knowledge scores and BMI stages (p=0.006) using the Kruskal-Wallis H test. This implies that there may be differences in knowledge levels across various BMI categories.

Table 4 presents an in-depth analysis of the relationship between various factors and complaints of vaginismus among the study participants. The chi-square test was employed to assess statistical significance. First, age appears to be a significant factor influencing complaints of vaginismus. Participants aged over 45 years had a higher proportion of complaints compared to younger age groups (1.0% vs. 5.6%, p=0.002). This suggests that older women may be more likely to experience vaginismus. Second, marital status also exhibited a strong association with vaginismus complaints. Single participants reported significantly lower complaints of vaginismus compared to married individuals (0.0% vs. 4.6%, p<0.001). This finding suggests that marital status may play a role in the occurrence of vaginismus. Third, BMI stages showed a significant association with vaginismus complaints (p=0.022). Notably, participants categorized as obese class 3 had a higher proportion of complaints compared to those in other BMI categories (0.4% vs. 1.4%, p=0.022). Lastly, health status was also related to complaints of vaginismus, with diseased participants reporting a slightly higher proportion of complaints (0.8% vs. 3.8%, p=0.042). It is important to note that while these associations are statistically significant, the overall prevalence of vaginismus remained relatively low among the study participants. 

**Table 4:** Relation of diseased women and different factors (N=500) χ2  test is significant if p≤0.05 and non-significant if p≥0.05.

Variables (frequency/percentage)	Do you complain of vaginismus?		X^2^	P-value
No	Yes
Age				
Less than 25 years	236	5	12.558	0.002
	47.2%	1.0%		
From 26 to 45 years	213	13		
	42.6%	2.6%		
Over 45 years	28	5		
	5.6%	1.0%		
Nationality				
Saudi	449	22	.093	0.609
	89.8%	4.4%		
Non-Saudi	28	1		
	5.6%	0.2%		
Marital status				
Single	267	0	27.749	0.0001
	53.5%	0.0%		
Married	209	23		
	41.9%	4.6%		
Level of education				
Illiterate	5	1	2.053	0.358
	1.0%	0.2%		
Secondary	99	5		
	19.8%	1.0%		
University	373	17		
	74.6%	3.4%		
BMI stages				
<18.5 underweight	49	0	13.097	0.022
	9.8%	0.0%		
18.5-24.9 normal weight	206	15		
	41.3%	3.0%		
25-29.9 overweight	151	4		
	30.3%	0.8%		
30-34.9 obesity class 1	55	2		
	11.0%	0.4%		
35-39.9 obesity class 2	8	0		
	1.6%	0.0%		
≥40 obesity class 3	7	2		
	1.4%	0.4%		
Health status				
Healthy	451	19	35.930	0.042
	90.2%	3.8%		
Diseased	26	4		
	5.2%	0.8%		
Age at the onset of menstruation				
Less than 11 years old	54	2	2.971	0.396
	10.8%	0.4%		
12-14	328	13		
	65.6%	2.6%		
15 17	83	7		
	16.6%	1.4%		
More than 17 years old	12	1		
	2.4%	0.2%		
Duration of the menstrual cycle				
Less than 21 days	347	16	1.058	0.589
	69.4%	3.2%		
21-25 days	110	5		
	22.0%	1.0%		
More than 35 days	20	2		
	4.0%	0.4%		
Is the menstrual cycle regular?				
No	116	3	1.538	0.161
	23.2%	0.6%		
Yes	361	20		
	72.2%	4.0%		
Number of days of menstrual blood				
Less than 3 days	18	3	4.686	0.096
	3.6%	0.6%		
From 3 to 7 days	413	18		
	82.6%	3.6%		
More than 7 days	46	2		
	9.2%	0.4%		
The amount of menstrual blood is abundant.				
Low	32	4	6.107	0.047
	6.4%	0.8%		
Medium	417	16		
	83.4%	3.2%		
Abundant	28	3		
	5.6%	0.6%		
Do you suffer from menstrual pain?				
No	47	2	1.725	0.631
	9.4%	0.4%		
Low	139	4		
	27.8%	0.8%		
Mild	199	12		
	39.8%	2.4%		
Severe	92	5		
	18.4%	1.0%		
A source of knowledge				
Nil	277	9	3.600	0.463
	55.4%	1.8%		
Social media	101	8		
	20.2%	1.6%		
Medical service providers	20	1		
	4.0%	0.2%		
Central media	17	1		
	3.4%	0.2%		
Friends and family	62	4		
	12.4%	0.8%		
How were you diagnosed?				
Nil	477	1	477.260	0.0001
	95.4%	0.2%		
Themself	0	7		
	0.0%	1.4%		
Doctor	0	15		
	0.0%	3.0%		

Table 5 focuses on identifying predictors of vaginismus among the participants using logistic regression analysis. Among the variables analyzed, age was a significant predictor of vaginismus complaints. Specifically, participants aged over 45 years had significantly higher odds of experiencing vaginismus compared to those aged 26-45 years (odds ratio=8.429, p=0.001). This suggests that older age is a risk factor for vaginismus. 

**Table 5:** Predictors of vaginismus among participants *Significant association Spearman's correlation. 95% CI: confidence interval; p-value: significant if p≤0.05 and non-significant if p≥0.05.

Variables		Odds ratio	95% CI*		P-value*
Lower	Upper
Age	From 26 to 45 years	2.881	1.010	8.215	.048
	Over 45 years	8.429	2.297	30.928	.001
Nationality	Non-Saudi	1.372	.178	10.552	.761
Level of education	Secondary	4.388	.486	39.658	.188
	University	1.108	.399	3.078	.844
Age at the onset of menstruation	12-14	.444	.037	5.310	.522
	15 17	.476	.057	3.938	.491
	More than 17 years old	1.012	.114	8.962	.991
Duration of the menstrual cycle	21-25 days	.461	.099	2.145	.324
	More than 35 days	.455	.082	2.507	.365
Is the menstrual cycle regular?	Yes	.467	.136	1.599	.225
Number of days of menstrual blood	From 3 to 7 days	3.833	.591	24.880	.159
	More than 7 days	1.002	.225	4.458	.997
Number of days of menstrual blood	Medium	1.167	.240	5.667	.848
	Abundant	.358	.098	1.302	.119
Do you suffer from menstrual pain?	Low	.783	.146	4.189	.775
	Mild	.529	.139	2.024	.353
	Severe	1.110	.380	3.242	.849
A source of knowledge	Social media	.504	.150	1.688	.266
	Medical service providers	1.228	.355	4.248	.746
	Central media	.775	.082	7.342	.824
	Friends and family	.912	.096	8.703	.936
BMI		1.009	.947	1.074	.789
Knowledge score		.986	.808	1.203	.889

No other demographic variables, such as nationality or level of education, were found to be significant predictors of vaginismus complaints. Additionally, variables related to menstrual characteristics, including age at the onset of menstruation, duration of the menstrual cycle, and the number of days of menstrual blood, were not significant predictors of vaginismus.

Similarly, factors, such as the regularity of the menstrual cycle, amount of menstrual blood, and presence of menstrual pain, were not significant predictors of vaginismus complaints. Furthermore, the source of knowledge about vaginismus and BMI were also not significant predictors of vaginismus. Lastly, the knowledge score regarding vaginismus did not emerge as a significant predictor of complaints about vaginismus among the study participants.

## Discussion

Vaginismus is a distressing and often misunderstood sexual dysfunction that can profoundly impact the physical and psychological well-being of women [16,17]. Understanding the prevalence and factors associated with vaginismus is crucial for healthcare professionals to provide appropriate care and support to affected individuals [4,18]. This cross-sectional study aimed to investigate the prevalence of vaginismus among women in Najran, Saudi Arabia, and assess their knowledge of this condition. The study revealed several noteworthy findings related to the study aim, shedding light on the epidemiology and awareness of vaginismus in this region.

One of the primary findings of this study is the prevalence of vaginismus among women in Najran, Saudi Arabia. The data showed that 23 participants (4.6%) of the surveyed women reported complaints of vaginismus. This prevalence rate aligns with existing literature on vaginismus, which suggests that it is a relatively rare condition compared to other sexual dysfunctions [15,19]. It is worth noting that the prevalence of vaginismus may be underreported due to the sensitive and stigmatized nature of the condition, as well as cultural factors that discourage open discussion of sexual issues.

Age emerged as a significant factor in the study's findings. Women aged over 45 years had a significantly higher prevalence of vaginismus complaints compared to those aged 26-45 years. This finding is consistent with previous research indicating that vaginismus may become more prevalent with age [20]. It is important to note that this age-related increase in prevalence may be attributed to various factors, including changes in hormonal levels, menopausal transitions, and psychological factors that accumulate over time [17,20].

Marital status was another demographic factor significantly associated with vaginismus. Married women reported a higher prevalence of vaginismus complaints compared to single women. This observation suggests that marital status could be a significant determinant of vaginismus, potentially related to relationship factors, sexual expectations, or cultural influences [18]. However, further research is needed to explore the complex interplay between marital status and vaginismus.

The study also investigated the relationship between BMI and vaginismus. While a statistically significant correlation was found between BMI and knowledge scores, the practical significance of this relationship may be limited. BMI categories did not emerge as significant predictors of vaginismus complaints. This finding indicates that factors other than BMI may play a more prominent role in the development or experience of vaginismus [21].

Health status was another variable examined, and it was found that women with pre-existing medical conditions reported a slightly higher prevalence of vaginismus complaints. However, the association was relatively weak. The relationship between health status and vaginismus warrants further investigation, as it may be influenced by a range of medical and psychological factors [18].

Assessing knowledge and awareness of vaginismus is a crucial aspect of understanding this condition. The study found that, on average, participants possessed a moderate level of knowledge about vaginismus. This level of awareness is encouraging, as it suggests that women in Najran, Saudi Arabia, have access to information about this condition. However, it is noteworthy that nearly 60% of participants reported having no source of knowledge about vaginismus. This underscores the importance of improving educational efforts and increasing awareness about sexual health issues in the region, especially given the sensitive and often hidden nature of vaginismus [22].

The logistic regression analysis revealed that age, particularly being over 45 years old, was a significant predictor of vaginismus complaints among the study participants. This finding aligns with previous research suggesting that older age may be associated with a higher risk of vaginismus [18,19]. However, it is essential to recognize that vaginismus is a complex condition influenced by various psychological, cultural, and interpersonal factors, and age alone does not determine its occurrence.

Other demographic factors, including nationality, level of education, and age at the onset of menstruation, did not emerge as significant predictors of vaginismus in this study [21]. Similarly, menstrual characteristics, such as cycle regularity, duration of menstrual cycle, and the amount of menstrual blood, did not show significant predictive relationships with vaginismus [17,22].

Limitations and future directions

This study contributes valuable insights into the prevalence and awareness of vaginismus among women in Najran, Saudi Arabia. However, several limitations should be acknowledged. The cross-sectional design limits our ability to establish causality or track changes over time. Additionally, the study relied on self-reported data, which may be subject to recall bias or social desirability bias, particularly concerning sensitive topics, such as sexual health.

Future research should explore the psychological and cultural factors influencing vaginismus in this population, as well as the impact of marital dynamics, sexual education, and access to healthcare services. Longitudinal studies could provide a deeper understanding of the factors contributing to the onset and persistence of vaginismus over time.

Recommendations

It is imperative that healthcare professionals, policymakers, and educators work together to provide comprehensive sexual health education and support to women in the region, with a focus on vaginismus.

## Conclusions

Vaginismus is a common disease that women suffer from and are shy to complain about to physicians. This study offers valuable insights into the prevalence and awareness of vaginismus among women in Najran, Saudi Arabia. The findings highlight the importance of age as a significant predictor of vaginismus complaints and underscore the need for increased awareness and education about this condition, particularly among older women. Education of patients about vaginismus and treatment methods lead to success treatments for them. It should be emphasized the early diagnosis of vaginismus will prevent psychiatric and sexual disorders in the future.
